# Work engagement and individual work performance in the UAE: the mediating role of work-life balance

**DOI:** 10.3389/fsoc.2025.1567207

**Published:** 2025-04-08

**Authors:** Mohamed A. Alnagbi, Hazem Aldabbas, Liza Gernal, Abdallah M. Elamin, Ahmed Z. E. Ahmed

**Affiliations:** College of Business Administration, University of Fujairah, Fujairah, United Arab Emirates

**Keywords:** work engagement, employee engagement, work-life balance, individual work performance, employee performance, UAE

## Abstract

This research focuses on how employees balance their engagement with their work and private life, and how this balance affects their performance in the workplace. The study conducted a survey completed by 98 employees from government, private, and non-profit organizations regarding their experiences with engagement, work-life balance, and performance. The findings indicate that workers in UAE-based workplaces are highly engaged in their jobs and perform significantly well. The key results show that work engagement is positively related to work-life balance, which, in turn, positively impacts individual work performance. Additionally, the study demonstrates that work-life balance mediates the relationship between work engagement and individual work performance, helping to explain and facilitate this relationship. This study provides a deeper understanding of work engagement and work-life balance in the UAE and offers insights into how organizations can implement best practices to enhance performance.

## 1 Introduction

In today’s competitive business environment, organizations should prioritize employee engagement and work-life balance to enhance performance and maintain a sustainable edge. Employee engagement, characterized by emotional and psychological commitment to the organization, is a critical driver of productivity and performance (Abdul-Azeez et al., 2024; Kahn, 1990). However, achieving high engagement requires addressing the challenges of maintaining a healthy work-life balance, as imbalances can lead to burnout and reduced performance (Greenhaus and Allen, 2011). This study explores the interplay between employee engagement, work-life balance (WLB), and individual work performance (IWP), aiming to provide actionable insights for organizations seeking to foster environments that support employee well-being while driving organizational success. Further, in this manuscript, the terms ‘work engagement’ and ‘employee engagement’ are used interchangeably to refer to the same concept. Similarly, the terms ‘individual work performance’ and ‘employee performance’ are also used interchangeably.

As employees strive to enhance their competitiveness, organizations should focus on fostering employee engagement to remain competitive. However, engagement is often linked to an employee’s ability to achieve a healthy work-life balance. This is particularly true since disengagement and engagement are influenced by individual priorities and life aspirations. Thus, balancing work and personal life has become increasingly challenging, especially in a society shaped by flexible work environments. These dynamics necessitate strong psychosocial coping mechanisms to manage the daily pressures of maintaining both work and home responsibilities. Such a phenomenon proves that organizations play an essential role in addressing this issue, Organizations really aim to attract and retain employees who are dedicated and engaged in their roles (Garg and Singh, 2020).

Findings from 37 publications suggest it is premature to conclude a dominant influence between variables. In 16 studies, WLB is more often explored as an antecedent of work engagement, while 12 studies show work engagement as a precondition for WLB. This indicates a bidirectional relationship rather than clear causality. Further longitudinal and meta-analytic studies are needed to clarify their causal links and quantitatively measure their associations (Wood et al., 2020). Given that the majority of these studies focus on Western contexts, our research extends the literature by examining this relationship in the UAE. The UAE is noted for its unique cultural and economic factors—such as high living costs, and evolving HR policies that may influence the work engagement and WLB dynamic differently. It is through understanding whether WLB serves as a driver or as an outcome of work engagement that makes this setting crucial at this stage. This study, therefore, builds upon existing literature by providing new insights into this relationship within the UAE’s organizational environment with the path direction from work engagement towards WLB. Moreover, high levels of work engagement empower employees with a clear sense of purpose and control over their schedules. This enables them to effectively allocate time for personal commitments, including family responsibilities. It is evident that engaged employees are more likely to approach their work with efficiency and focus. This approach ultimately reduces unnecessary stress and therefore allow for a more structured and balanced integration of professional and personal life.

Organizations in the UAE face significant challenges in fostering employee engagement, which is crucial for organizational success. Limited resources and high employee turnover create barriers to maintaining a committed and motivated workforce. When employees are not effectively engaged, their connection to the organization weakens, leading to decreased productivity and retention issues. This therefore raises a critical concern about how organizations can enhance employee engagement to drive better performance outcomes. Furthermore, WLB plays a mediating role in this relationship, influencing the extent to which engagement translates into improved performance. Long working hours, rigid organizational structures, and uneven workloads contribute to stress and burnout. As a consequence, this negatively affect both engagement and overall job effectiveness. The inability to create a supportive WLB environment may weaken the link between engagement and performance, making it essential to explore strategies that optimize both employee well-being and organizational outcomes. Thus, the research problem centers on understanding the relationship between employee engagement and IWP in organizations in the UAE, with WLB as a mediating factor. This study aims to identify key drivers of engagement and assess how work-life balance influences the impact of engagement on IWP.

Many recent studies report on a positive relationship between work engagement and employee creativity, innovation, and performance (Elamin et al., 2024b; Garg and Singh, 2020; Nusraningrum et al., 2024). However, research on the interplay between engagement, work-life balance, and job performance remains limited, particularly in the context of the UAE and the broader Middle East. This gap highlights the need for empirical studies that can inform HR policies and practices, ensuring they are aligned with the realities of the workplace. By addressing these gaps, this study further aims to provide actionable insights that can enhance both employee and organizational performance in the UAE and beyond. While extensive research has explored employee engagement in Western countries, there remains a significant gap in studies focusing on the Middle East, particularly in the UAE (Elamin et al., 2024b).

The empirical study is conducted in the UAE, recognized as one of the most advanced nations in the Arab world (Aldabbas et al., 2025). Additionally, the UAE presents a unique and relevant context for studying WLB due to several distinct challenges faced by employees in the region as summarized by (Bhatnagar, 2025). One of the primary concerns is the prevalence of long working hours, which limits employees’ ability to engage in personal and family life. The country’s highly competitive work culture further intensifies job-related stress. Such culture really makes it difficult for employees to disconnect from work as well as maintain a healthy balance between professional and personal responsibilities. While many UAE-based companies provide attractive compensation and benefits, formal policies that facilitate flexible work arrangements, reduced working hours, or structured time-off remain insufficient. This lack of institutional support can make it difficult for employees to manage their personal and professional commitments effectively.

Given these factors, the UAE serves as a compelling setting for examining the role of WLB in employee engagement and IWP. Investigating WLB in this context provides valuable insights into how organizations can develop better policies to enhance employee engagement, and performance, ultimately contributing to more sustainable work environments in the region. UAE through its labor law, aims to create optimal working conditions across both the private and public sectors (e.g., WLB) (Adekoya et al., 2021). Thus, beginning in 2022, the government of the UAE implemented a 4.5-day workweek to enhance work-life balance. Consequently, the aim of this study is to examine the relationship between employees’ engagement and their work-life balance, as well as whether WLB would mediate this relationship between work engagement and IWP. Our goal is therefore to answer the following research questions: Is there a relation between work engagement and individual performance at work? If so, does WLB mediate the relationship between work engagement and IWP? The theoretical framework and literature are discussed further.

## 2 Theoretical framework and literature review

### 2.1 Social Echange theory

This study adopted Social Exchange Theory (SET) which is widely regarded as a significant framework for examining employee behavior in the workplace, due to its adaptability in analyzing both direct and indirect relationships within a unified conceptual model (Aldabbas et al., 2025). Since individuals and groups may respond differently based on their exchange ideology, this results in employees having low exchange ideology. Thus, they are less likely to focus on obligations or feel concerned when exchanges are not reciprocated. Conversely, those with a strong exchange ideology are more inclined to reciprocate positive actions compared to individuals with a weaker exchange ideology. Consequently, engaged employees not only perform better but also contribute to higher profitability and talent retention, creating a mutually beneficial relationship between employees and employers (Saks, 2022; Aldabbas and Blaique, 2025).

Academic and industry research have made rapid progress on employee engagement and WLB as discrete fields of study. This progress, however, has far outpaced our understanding of the connectivity between the two. Engaged employees may have better work-life balance, and yet people who perceive that they have a good WLB enhance her performance. The academic literature, however, has been reluctant to commit resources to understanding the relationship between engagement, work-life balance, and IWP. This work will also help to identify policy decisions that may need to be taken as a result of the evidence, and as they cascade down into the workplace.

The researchers suggest that work engagement can promote a better WLB for employees (Ali et al., 2022). Our study conceptualizes work engagement as a precursor to WLB based on SET. The idea is that when an employee feels their work and engagement on certain days of the week are valued by the organization, they can expect their manager to consider this if the employee needs extra break time or has a critical family matter. The employee understands that their direct manager will justify this request, recognizing their commitment and engagement on other days of the week. Hence, the relationship between employees and employers is grounded in social exchange principles, where employees are motivated to reciprocate care and consideration (Saks and Gruman, 2024). Thus, this approach will help keep the employee motivated and engaged in organizational activities.

### 2.2 Work engagement

The expression ‘employee engagement’ has been used in literature as a synonym of employee involvement, organizational commitment, job satisfaction, employee satisfaction and employee participation. Even though there are some overlaps, many scholars have advanced differently, or perhaps have multiple, definitions of this notion which vary in respect of focus and emphasis. These definitions share characteristic features, namely, engagement as considered to be one of the key drivers of effectiveness. Such key drivers involve such goals as the following: job performance and organizational citizenship, retention, identification with and loyalty to the company, and readiness to ‘stretch’ when required. Employees who are engaged, therefore, possess ‘vigor’, a tendency to expend energy in working towards reaching personal work goals and also ‘absorption’ in the goal of making work a part of one’s life. Engagement is associated with energy and positive outlook, and therefore they are widely considered to be a critical factor in productivity and performance. An employee engaged in the work gets oriented as well as psychologically attached to both personal and organizational objectives. They are known to exert extra time and effort leading to heightened productivity and employee’s performance (Clack, 2021).

Engagement, on the other hand, depends upon the culture of the organization as well as the leadership style within the organization, implying that some strategy will have to be devised to address the issue. According to another widely used model, the job demands-resources model Bakker and Demerouti (2014) views the interaction between the demands in the emotional and physical ‘job demands’ and the ‘resources’ that are available. In this case, positive attitudes, work behaviors, and intentions are the consequences of high job resources; well-being is inversely influenced by ‘impact on work being resourceful’. The individual’s perception of whether the demands and resources are at the right level results from assessments of resources and demands. This leads to the withdrawal of the individual’s resources, resulting in burnout if there are more demands within the physical and psychological demands than there are resources (Demerouti and Bakker, 2023).

### 2.3 Work-life balance

WLB has gained significant attention in recent years (Jaharuddin and Zainol, 2019). It refers to an individual’s ability to efficiently allocate time and energy to fulfill work responsibilities, personal needs, leisure activities, and family obligations (Susanto et al., 2023). The literature on work-life initiatives is extensive. However, the terms work-life “balance” and “work-life programs” cover a wide spectrum of issues. Our initial task, therefore, was to address the issue of what is commonly referred to as work-life balance. This is WLB referring to both work roles and home, or family life being in equilibrium (Greenhaus and Allen, 2011). Furthermore, Haar et al. (2014) highlights how employees having “work-life balance” have less stress and high job satisfaction.

Research indicates that WLB initiatives are helpful in attracting and retaining employees, and in maintaining high levels of workforce morale and creativity as emphasized by Deery (2008). The implementation of work-life programs and practices can be used as a strategic lever for achieving enhanced organizational performance. Many organizations have implemented schemes to support work-life balance. For example, flexible working arrangements offer people a means of tailoring their work conditions to specifically suit their personal and/or family needs such as working remotely (Jiang et al., 2023).

## 3 Research hypotheses development

### 3.1 The relationship between work engagement and individual work performance

A supportive and positive work environment significantly enhances employee engagement, which in turn boosts organizational performance by improving morale and increasing productivity (Elamin et al., 2024a). In addition, high levels of engagement can also improve the levels of trust employees have in management. The engagement of employees translates into positive workplace behaviors that lead to the success of an organization (Saks, 2006, 2022).

Sopian et al. (2022) find positive and significant relationship between employee engagement and IWP for 211 participants in Indonesia. Additionally, a survey of 383 employees found that engagement corelates positively with employee performance (Anitha, 2014). In like manner, a survey of 222 employees in the logistics sector revealed that work engagement significantly enhances employee performance (Nusraningrum et al., 2024). Furthermore, an empirical study for 369 IT developers in India found that work engagement positively and significantly influences IWP (Garg and Singh, 2020).

SET suggests that when employees feel appreciated and supported by their organization, they are more inclined to respond with greater engagement and dedication (Cropanzano and Mitchell, 2005). Thus, engaged employees, driven by mutual trust and a sense of obligation, are more likely to put additional effort into their tasks, resulting in enhanced performance. This reciprocal dynamic cultivates a positive workplace atmosphere where employees are motivated to contribute to organizational success. Consequently, engagement serves as a key driver of performance, fostering a cycle of mutual benefit between employees and their organizations. Based on the previous arguments, the first hypothesis is formulated.

*H1*: Work engagement positively influences individual work performance

### 3.2 The relationship between work engagement and work-life balance

Work-life balance has a significant impact on employee engagement (Wood et al., 2020; Žnidaršič and Bernik, 2021). Creating a healthy balance can be achieved by providing employees with autonomy over their roles, trusting them to do their jobs, and engaging them frequently (Wood et al., 2020). This includes offering opportunities for healthy communication in the workplace (Wood et al., 2020). On the other hand, when employees are engaged in their work and have a good work-life balance, it can have positive effects on various aspects of their performance, including productivity and job quality (De Kort and Poell, 2016). Haar et al. (2017), also found the effect of WLB on engagement. However, a sample from 372 senior employees in China across the IT, trade, real estate, financial, and telecommunications sectors revealed that highly engaged employees are more likely to maintain a good WLB (Ali et al., 2022).

Overall, the research suggests that work engagement positively influences WLB, and vice versa. By promoting a healthy WLB and providing opportunities for employee engagement, organizations can create a positive work environment that benefits both employees and the company as a whole. We suggest that when employees are actively engaged in their tasks during workdays, it positively impacts their well-being and satisfaction with their roles. This engagement reinforces their perception of being valuable resources to the organization. Moreover, such engagement provides employees with the clarity and opportunity to thoughtfully manage their time and prioritize life considerations, such as family responsibilities. This perspective aligns with SET, as employees who feel valued and supported through engagement are more likely to reciprocate by effectively managing their WLB. Based on this argument, the second hypothesis is formulated:

*H2*: Work engagement positively influences work-life balance.

### 3.3 The relationship between work-life balance and individual work performance

Work-life balance plays a crucial role in enhancing both the quality of life for employees and the overall effectiveness of organizations (Bataineh, 2019). By fostering an environment that supports flexibility and well-being, organizations can empower their workforce to manage personal and professional responsibilities more effectively. This balance not only leads to greater employee satisfaction but also contributes to improved performance and productivity, creating a win-win scenario for both employees and employers.

The literature in this area postulates that WLB practices improve not only employee well-being but also act as a catalyst to improve performance metrics within organizations. Beauregard and Henry (2009) provided basic understanding of this relationship by illustrating that the more WLB practices are available, the better the productivity and superior management practices in medium-sized manufacturing firms across the USA and Europe. Salolomo and Agbaeze (2019) address how WLB relates to employee performance in Nigerian banks. According to this study, the influence of the WLB leads to higher performance. Udin (2023), find a positive relationship between WLB and employee performance. A study of 289 employees from the pharmaceutical industry in Jordan demonstrated that WLB positively influences IWP (Bataineh, 2019). A study conducted with 357 administrative staff in Malaysia revealed a positive correlation between WLB and employee performance (Abdirahman et al., 2018). There is a significant interlinkage between WLB practices and organizational performance, according to (Beauregard and Henry, 2009). They note that organizations offering flexible work arrangements, such as flexible hours and telework, enjoy increased productivity and lower turnover rates. The authors also contend that such arrangements evoke reciprocity, whereby employees try harder during peak periods, in return for the flexibility they enjoy. In the context of SET, the social interactions between employees, and their supervisors facilitated through participation in training programs and the implementation of flexible working hours and work engagement. This flexibility enables employees to choose schedules that best suit their family needs while ensuring adequate breaks during work (Bataineh, 2019). Based on the previous argument, the third hypothesis is formulated:

*H3*: Work-life balance positively influences employee performance.

### 3.4 The mediating role of work-life balance

This section examines the relationship between work engagement and IWP through the mediating role of WLB. However, WLB programs aim to assist employees in managing the responsibilities of both their professional and personal lives while reducing and addressing conflicts between work and family roles (Saks, 2022). Thus, the concept of WLB directly affects both the performance of an organization and its employees. It involves managing how and when individuals experience and perceive this balance (Sreya et al., 2023). When discussing the mediating role of WLB, much of the focus has been on the impact of organizational policies designed to support employees. These policies aim to reduce the conflicts that arise between employees’ professional responsibilities within the organization and their personal lives, including their roles at home and within society (Tamunomiebi and Oyibo, 2020).

Data was gathered from 334 employees employed across various private sector organizations in Pakistan, revealing that WLB mediates the connection between employee engagement and IWP (Riaz et al., 2021). Data from 452 nursing professionals in India during the COVID-19 pandemic revealed that job resources and WLB had a positive impact, which, in return, was associated with higher job satisfaction among nursing professionals (Rashmi and Kataria, 2023). A sample of 316 from a study population of 2,045 administrative staff at a public university in Ghana found that WLB significantly mediated the relationship between flexible work arrangements and employee performance (Eshun and Segbenya, 2024).

SET provides a logical framework for understanding the interdependence between employees and organizations (Elamin et al., 2024a). SET suggests that employees who perceive strong organizational support feel a sense of obligation to reciprocate by demonstrating positive attitudes and behaviors (Kuvaas et al., 2020). According to this theory, when employees perceive that their employer supports them through WLB initiatives, they are more likely to reciprocate by enhancing their work engagement, putting greater effort into their job performance, and contributing to the organization’s success. Based on the previous argument, the final hypothesis is thus formulated:

*H4*: Work-life balance mediates the relationship between work engagement and individual employee performance.

**Figure 1:**
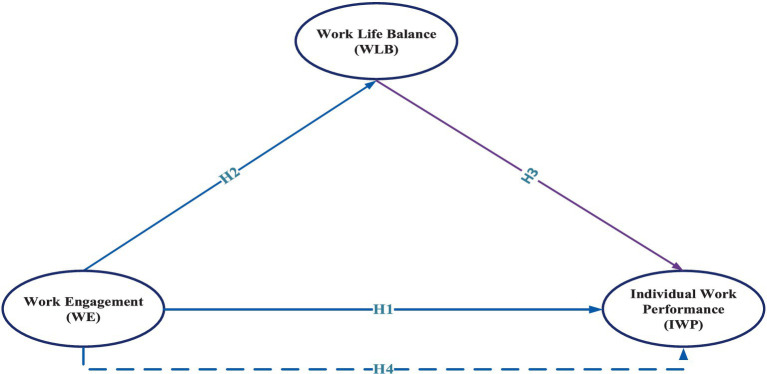
Hypothesized model.

This conceptual framework attempts to examine the relationship between work engagement, WLB and IWP. Such model implies that if the WLB is maintained, employees will be engaged and employees’ performance will be high. It also posits that work-life balance may explain the relationship between work engagement and IWP (Figure 1).

## 4 Methodology

### 4.1 Research design

The purpose of this research is to investigate the relationship between work engagement, work-life balance, and individual work performance in UAE organizations. As such, employing the *quantitative research approach* serves as a convincing method for studying the relationships between the main variables of the study. However, all the data has been collected through a constructive survey instrument.

### 4.2 Participants and data collection

This study employed a convenience sampling method, a cost-efficient and time-effective approach to reaching potential participants (Dillman et al., 2014; Etikan et al., 2016). In detail, the survey was distributed to approximately 200 employees working in government, private companies, and non-profit organizations. This study specifically targets full-time employees who have been with their organization for at least 6 months, ensuring that participants can provide informed opinions on key variables such as work engagement and WLB. Employees with less than 6 months of tenure may not have sufficient experience to accurately assess these variables. Therefore, part-time workers, freelancers, and employees with less than 6 months of tenure are not included in the study.

The survey was conducted using Google Forms, which includes a built-in consent form. Various techniques have been employed to collect data. Participants received an invitation email containing the survey link or were contacted through professional networks and LinkedIn groups via private messages. Before sending the survey, it is ensured that the recipients are employees working in organizations in the UAE and have been in their roles for at least 6 months. Incomplete responses were excluded from the final dataset.

The appropriate sample size was determined as crucial for ensuring the validity and reliability of survey research. According to Bartlett et al. (2001), for a population size of 200, aiming for a 95% confidence level with a margin of error of 0.03, the recommended sample size is approximately 75 respondents. If the researcher seeks a significance level (α) of 0.01, with t = 2.58, the required sample size increases to 102 respondents. However, the data collection period spanned 2 weeks in January 2025, with weekly reminders sent to enhance response rates. The study consists of a sample size of 98 participants, resulting in a 49% response rate. The target sample size aligns with guidelines for correlational research, which recommend at least 85 participants, and for multiple regression analysis, where a range of 85 to 120 participants is suggested to achieve adequate statistical power, as per Cohen’s recommendations (Chuan and Penyelidikan, 2006).

### 4.3 Measurements

Work Engagement represents the independent variable in this study. Employee engagement is the area of employees’ physical and emotional ties related to their job. We adopted employee engagement scale which is developed by Schaufeli et al. (2006), UWES-9 developed by Schaufeli and his colleagues looked into three dimensions, i.e., vigor, dedication and absorption: an example of an item includes “I feel energized at work” (vigor) and “I am enthusiastic about my job” (dedication). Participants express themselves through a 7-point Likert’s scale: where 1 = strongly disagree, and 7 = strongly agree.

Work Life Balance Scale (WLB) represents the mediator variable. This variable refers to the employees’ skill in combining professional and personal obligations as dictators of their commitment. WLB scale was adapted from Carlson et al. (2000). Example of items in this scale is ‘The demands of my work interfere with my family responsibilities.’ All responses are obtained using a 5-point Likert scale (1 = strongly disagree, 7 = strongly agree).

Individual Work Performance (IWP) represents the dependent variable. These variable measures employee’s task performance, contextual performance and counter productive work behavior which is adapted from Widyastuti and Hidayat (2018). Examples are: “I never fail to meet my deadlines for work” and “I contribute to a positive work environment.” All responses are given on a seven-point Likert scale. Details of the survey items can be found in Appendix A.

## 5 Data analysis and results

### 5.1 Demographic variables

The demographic variables are considered as gender, age, sector, job role and years of experience. This will offer insight into general trends and patterns that characterize the dataset and assist in forming an understanding of the general basic characteristics of the respondents.

Table 1 illustrates that the sample consists of 38 females (39%) and 60 males (61%). The majority of respondents (50%) are aged 18–25 years. Additionally, 34.7% fall within the 26–35 age group. The remaining 15.3% are 36 years and older. The employment sector has the largest share of respondents, constituting 74%, works in the government sector, followed by 12.1% in both private organizations and non-profit sectors. As for Job Role, it shows that the largest group of respondents (37.5%) is employees in the middle level range, while the rest have entry level positions at 25%, senior level positions at 21.9 percent, and 15.6 percent in top management positions. The data suggests there is a relatively equal distribution of employees across all these volunteers career progression. Years of Work Experience: The number of youth respondents who have less than a year of experience is only 12.1, whereas around half, or 55.7 of them have between 1 to 10 years’ worth of experience.

**Table 1:** Demographics of respondents.

Demographic category	Number of respondents	Percentage
Gender		
Female	38	39%
Male	60	61%
Age		
18–25	49	50.0%
26–35	34	34.7%
36 and above	15	15.3%
Employment Sector		
Government	74	69.7%
Private	12	12.1%
Non-profit	12	12.1%
Job role		
Entry-level	25	25%
Mid-level	38	37.5%
Senior-level	22	21.9%
Executive/Leadership	16	15.6%
Years of experience		
Less than 1 year	12	12.1%
1–3 years	21	21.2%
4–7 years	21	21.2%
8–10 years	18	18.2%
More than 10 years	27	27.3%

### 5.2 Common method variance

Two practices were followed to ensure that CMV is not an issue in this study. First, all factors were merged (work engagement, WLB, and IWP) and the result shows a not-fit model, like a comparative fit index (CFI) = 0.624; Tucker–Lewis index (TLI) = 0.584; the standardized root mean square residual (SRMR) = 0.162; and, the root mean square error of approximation (RMSEA) = 0.260. While the proposed (hypothesised mode) reports the following results: (CFI = 0.961, TLI = 0.953, SRMR = 0.056, RMSEA = 0.090). Thus, since the fit of the model with all factors combined is poorer than that of the proposed model, CMV is not considered a concern (Podsakoff et al., 2024). Second, Harman’s single-factor test was conducted, following the approach outlined by Podsakoff et al. (2003). Using an unrotated factor analysis, the results indicated that the first factor accounted for 39.795% of the variance, which is well below the 50% threshold. This finding suggests that CMV was not a significant concern in this study.

### 5.3 Correlation analysis

Pearson’s correlation (r) analysis revealed several key relationships between the key variables (Table 2). First, work engagement and IWP received the highest correlation (r = 0.674). Second, work engagement and WLB, indicates positive work engagement correlates positively with WLB (r = 0.639). Lastly, WLB and IWP was found to be positively correlated (r = 0.634). Additionally, Table 2 shows the mean and standard deviation for the main variables.

**Table 2:** Correlation (r), mean, and standard deviation.

Variables	Work engagement	Work-life balance	Individual work performance	Mean	Std. Deviation
Work engagement	1			4.150	2.095
Work-life balance	0.639^**^	1		3.002	1.300
Individual work performance	0.674^**^	0.634^**^	1	3.332	0.909

** Correlation is significant at the 0.01 level (2-tailed).

### 5.4 Analysis of variance

An ANOVA test was conducted to determine whether there is a significant difference between employees working in the public, private, or non-profit sectors. The results indicate no significant differences between employee sector (public, private, or non-profit) and WLB (*F* = 0.269, *p* = 0.765). Same as between employee sector and IWP (*F* = 0.599, *p* = 0.551). As a result, ANOVA indicated no significant differences between demographic factors (e.g., the three different sectors). Consequently, these factors were excluded from further analysis, as controlling for them was deemed unnecessary due to their lack of impact on the primary variables in our dataset (Aldabbas and Bettayeb, 2024). This may refer to UAE having a Vision strategy that aims to position the country among the world’s leading nations by aligning with pioneering programs and projects in governmental development, service enhancement, and innovation. While many organizations, whether governmental or private, choose to adopt existing excellence models to foster and improve their standards, the UAE leadership has taken a proactive approach by creating a comprehensive excellence framework representing a new evolution in quality management (Salah and Salah, 2019). Moreover, the government of the UAE is known for implementing best practices in governance, public service, and infrastructure, often drawing inspiration from the luxury and hospitality sector. One notable example is the “Seven-Star Hotel Service Approach,” which reflects a commitment for the UAE government to excellence, efficiency, and customer satisfaction in public administration (Al Zaabi, 2019).

### 5.5 Hypothesis testing

The analysis of data will be carried out by using Statistical Package for the Social Sciences (SPSS). This is a very popular statistical analysis software package which is extensively used for social sciences research. It will allow for carrying out correlation analysis, hypotheses testing, and data visualization. Specifically, the hypotheses tested by using Process Macro (Model 4) (Hayes, 2018).

First, Hypothesis 1 was supported as the relationship between employee engagement and IWP was found to be both positive and significant (B = 0.197, t = 4.946, *p* < 0.001). Second, Hypothesis 2 was confirmed as there was a positive and significant direct relationship between employee engagement and WLB (B = 0.397, t = 8.141, *p* < 0.001). Third, Hypothesis 3 was confirmed by the significant and positive association between WLB and IWP (B = 0.240, t = 3.736, *p* < 0.001). Fourth, the mediation hypothesis was supported, with a positive and significant indirect relationship between employee engagement and IWP mediated by WLB (B = 0.095, bootstrapping SE = 0.030, *p* < 0.05). As a result, the indirect effect of employee engagement on IWP through WLB was both positive and significant. The adjusted R-squared value (R^2^ = 52.5%) indicates that employee engagement and WLB explain a significant portion of the variance in the dependent variable (IWP). Moreover, 40.8% of the variance in the mediator (WLB) was explained by employee engagement. Table 3 presents the results of the direct path coefficients and R-squared values.

**Table 3:** Regression coefficients, standard errors and model summary information.

Antecedents	M (Work-life balance (WLB))					Y (individual work performance (IWP))				
	B	SE	*t*	*p* value	[LLCI, ULCI]	B	SE	*t*	*p* value	[LLCI, ULCI]
EE	0.397	0.049	8.141	<0.001	[0.300, 0.493]	0.197	0.040	4.946	<0.001	[0.118 0.277]
WLB	-	-	-	-	-	0.240	0.064	3.736	<0.001	[0.113, 0.368]
Constant	1.356	0.226	5.994	<0.001	[0.907, 1.805]	0.1791	0.167	10.720	<0.001	[1.460, 2.123]
	R^2^ = 0.408					R^2^ = 0.525				
	*F* (1,96) = 66.273, *p* < 0.001					*F* (2,95) = 52.440, *p* < 0.001				

EE, employee engagement; WLB, work-life balance; IWP, individual work performance; SE, Standard Error; LLCI, lower limit confidence interval; ULCI, upper limit confidence interval.

## 6 Discussion, implications, and conclusion

### 6.1 Discussion of main findings

This study examines the relationship between employee engagement and employee performance. Additionally, it examines the mediating role of WLB in the relationship between employee engagement and IWP. The first hypothesis investigates the direct relationship between employee engagement and IWP. This is consistent with the previous studies which states that employees who are engaged were the ones that are likely to be active, more productive and will be able to help the organization as a whole (Saks, 2006). Additionally, high levels of engagement are consistent with previous studies and surveys, which highlight a positive association between engagement and achievement in relation to IWP (Anitha, 2014; Garg and Singh, 2020; Nusraningrum et al., 2024; Sopian et al., 2022). On the contrary, the negative impact of WLB on performance is a significant finding. For instance, employees who report high work-life interference tend to experience increased stress, lower job satisfaction, and decreased performance (Pattusamy and Jacob, 2017). Moreover, low employee engagement can lead to high turnover, poor customer service, and lower revenues and profitability (Tamunomiebi and Oyibo, 2020).

The second hypothesis examines the relationship between employee engagement and WLB. The findings of this study align with those research that determined employee engagement as a critical driver of WLB (Ali et al., 2022; Culbertson et al., 2012; Gaur, 2024). Additionally, work engagement affects WLB, as more vigor put into a job helps maintain better balance (Lee and Lee, 2023). As highlighted earlier, this study conceptualizes work engagement as antecedent to WLB, drawing on SET. The idea is that when employees feel their work and engagement are appreciated by the organization, they develop an expectation that their manager will reciprocate. For instance, if an employee requires additional break time or has an urgent family obligation, they trust that their manager will accommodate these needs, recognizing their dedication and contributions on other past days. This reciprocal relationship fosters motivation and sustained engagement in organizational activities. Additionally, the results are consistent with Ahmed et al. (2024) arguments that SET and reciprocity suggest that employees view their relationship with the organization as a mutual exchange of resources, responding in kind to the support they receive. When organizations provide employees with opportunities for development, and work engagement, employees are more likely to reciprocate by maintaining a healthier WLB. Higher engagement fosters a sense of commitment and job satisfaction, enabling employees to manage their professional and personal responsibilities more effectively.

The third hypothesis examines the relationship between WLB and employee performance. Our results are consistent with previous studies (Abdirahman et al., 2018; Bataineh, 2019; Beauregard and Henry, 2009; Salolomo and Agbaeze, 2019; Udin, 2023). Lastly, the results of this study are consistent with the recent study by Riaz et al. (2021).

The fourth and final hypothesis examined the relationship between work engagement and IWP through the mediating role of WLB. The results confirmed that WLB mediates the relationship between job resources and higher employee satisfaction (Rashmi and Kataria, 2023). Moreover, this study aligns with previous research that examines the mediating role of WLB in the relationship between flexible work arrangements and employee performance (Eshun and Segbenya, 2024). Our findings likewise align with a study that explored how WLB serves as a significant mediator in the relationship between work engagement and employee performance (Riaz et al., 2021).

### 6.2 Practical implications

This research reveals some fundamental aspects for the organizations that operate in the UAE. To manage employee engagement, WLB and performance management pose career developmental challenges as well as cross-sectoral ones.

The following are practical implications:

- Organizations should implement flexible work arrangements, such as remote work, compressed workweeks, and flexible hours, to help employees maintain a balance between work and personal life. This can enhance job satisfaction and reduce burnout, leading to higher engagement and performance. For instance, managers in the UAE should continue implementing initiatives that promote work-life balance, as these efforts have been shown to benefit employees and improve their overall performance (Allen, 2013; Bupa Global, 2024). By fostering policies such as flexible work arrangements organizations can create a supportive work environment that enhances employee performance.
- Managers should focus on employee engagement as a key driver of success. This involves creating opportunities for growth, aligning organizational goals with individual objectives, and overall performance. Thus, organizations should invest in training programs that focus on time management, stress reduction, and personal effectiveness. Employees who learn to balance work and life commitments effectively are more likely to demonstrate high performance. By doing so, managers can strengthen employee engagement, which positively impacts both individual and organizational performance (Macey and Schneider, 2008).
- Managers should recognize that employee engagement, work-life balance, and performance management are interconnected issues that go beyond conventional practices. Addressing these challenges requires innovative and integrated approaches that consider the unique needs of diverse sectors within the UAE (Murthy and Gernal, 2024). Thus, managers need to implement smart performance management practices by adopting flexible approaches that enable employees to engage effectively and maintain a healthy work-life balance.

### 6.3 Limitations and recommendations for future research

To enhance our understanding of the complex interrelationships among employee engagement, WLB, and IWP, future research directions are essential. These efforts can provide deeper insights into these constructs and their dynamic interactions within organizational settings. First, this study relies on cross-sectional and convenience sampling, providing a snapshot of these constructs at any one point in time. Engagement, WLB, and performance are dynamic and fluctuate within organizations for many factors, such as organizational changes, economic shifts, or personal situations. Longitudinal studies would thus enable researchers to track these variables over time and gain greater insight into how they evolve and interrelate. These might show long-term trends, causes, and effects of an intervention which will help organizations with continued improvement in engagement and performance. Second, this work relies on self-reports for individual work performance, which may introduce bias. Future studies could address this by incorporating supervisor or peer ratings to assess performance more objectively. Additionally, the small sample size of only 98 respondents limits the generalizability of the findings. Future research should consider expanding the sample size and employing random sampling techniques to enhance the reliability and applicability of the results. Third, future research needs to incorporate the unique cultural features of the UAE, as well as those of the Middle East more generally. In fact, in this region, a set of cultural norms and values often guide expectations about work behaviors and shape certain organizational practices. For instance, the focus of family or social relationship issues in Middle Eastern cultures may influence how people in such contexts view WLB. Fourth, there is a pressing need for research on how cultural factors influence employee engagement, WLB, and IWP in the UAE and the Middle Eastern countries that would be immensely helpful for organizations looking to develop culturally suitable policies and practices. Nevertheless, these cultural shades will form the basis for potentially effective strategies that resonate with employees in their work and consequently improve organizational performance. Finally, qualitative research methods, such as in-depth interviews or focus groups, might formulate a more complete overview of employees’ subjective experiences relative to engagement and work-life balance. Quantitative data show broad patterns; qualitative techniques go beyond the possibility of engaging personal stories and insights that quantitative measures cannot show. These methods could also explain the emotional and psychological aspects of engagement and WLB, thus helping to emphasize how workers really feel concerning their work atmosphere, challenges pertaining to private-professional life balance, and what impact these have on their overall performance. This deeper, more comprehensive understanding may be important in devising interventions that actually improve employee welfare and productivity.

### 6.4 Conclusion

This research focused on examining the link between employee engagement, WLB and job performance within the UAE organizations. The research sought to understand the link between these factors and their effects on job performance. The results that came out of the analysis were based on the sampled 98 employees in different sectors of companies in UAE. In conclusion, this research sheds light on the two-way relationship between employee engagement and WLB in the context of the UAE. The study shows that employee engagement is a concept which should receive close attention from management and explains the reason why relevant policies that promote WLB in the organization are necessary. Use of flexible work schedules and family-friendly policies could mitigate work–family conflict and increase employee and organizational effectiveness. All relationships studied should continue to be investigated in different types of employment and fields of business, especially in the United Arab Emirates or may elsewhere. Ultimately, organizations that have a culture of engagement and WLB will likely enhance employee performance.

## Data Availability

The raw data supporting the conclusions of this article will be made available by the authors, without undue reservation.

## Appendix A

### Work Engagement

The Utrecht Work Engagement Scale (UWES-9), adopted from Schaufeli et al. (2006) (7-point Likert scale: 1 = Strongly Disagree, 7 = Strongly Agree).

1. I feel energized at work.
2. I am enthusiastic about my job.
3. I feel happy when I am working intensely.
4. My work inspires me.
5. When I get up in the morning, I feel like going to work.
6. I am fully immersed in my job.
7. I feel strong and vigorous during my workday.
8. I am proud of the work that I do.
9. Time flies when I am working.

### Work-Life Balance (WLB)

The WLB Scale adapted from Carlson et al. (2000) (7-point Likert scale: 1 = Strongly Disagree, 7 = Strongly Agree).

1. The demands of my work interfere with my family responsibilities.
2. I often have to miss important personal activities because of work.
3. The time I devote to work keeps me from participating in personal interests.
4. My personal life interferes with my job responsibilities.
5. My family demands prevent me from giving my best at work.
6. The demands of my personal life make it difficult to focus on my job.

### Individual Work Performance (IWP)

The IWP scale adapted by Widyastuti and Hidayat (2018) (7-point Likert scale: 1 = Strongly Disagree, 7 = Strongly Agree).

1. I consistently meet my work deadlines.
2. I achieve the goals set for my job.
3. I take the initiative to solve problems at work.
4. I help colleagues when needed.
5. I contribute to creating a positive work environment.
6. I sometimes engage in behavior that could negatively impact my work. (Reverse Scored).
7. I make mistakes that affect my performance. (Reverse Scored).
